# A Novel Loss-of-Function Variant in Transmembrane Protein 263 (TMEM263) of Autosomal Dwarfism in Chicken

**DOI:** 10.3389/fgene.2018.00193

**Published:** 2018-06-05

**Authors:** Zhou Wu, Martijn F. L. Derks, Bert Dibbits, Hendrik-Jan Megens, Martien A. M. Groenen, Richard P. M. A. Crooijmans

**Affiliations:** Animal Breeding and Genomics, Wageningen University & Research, Wageningen, Netherlands

**Keywords:** autosomal dwarfism, body size, recessive trait, chicken, loss-of-function mutation

## Abstract

Autosomal dwarfism (adw) in chickens is a growth deficiency caused by a recessive mutation. Characteristic for adw is an approximately 30% growth reduction with short shank. The adw variant was first recognized in the Cornell K-strain of White Leghorns, but the genetic causal variant remained unknown. To identify the causal variant underlying the adw phenotype, fine mapping was conducted on chromosome 1, within 52–56 Mb. This region was known to harbor the causal variant from previous linkage studies. We compared whole-genome sequence data of this region from normal-sized and adw chickens in order to find the unique causal variant. We identified a novel nonsense mutation NP_001006244.1:p.(Trp59^∗^), in the transmembrane protein 263 gene (*TMEM263*), completely associated with adw. The nonsense mutation truncates the transmembrane protein within the membrane-spanning domain, expected to cause a dysfunctional protein. *TMEM263* is reported to be associated with bone mineral deposition in humans, and the protein shows interaction with growth hormone 1 (GH1). Our study presents molecular genetic evidence for a novel loss-of-function variant, which likely alters body growth and development in autosomal dwarf chicken.

## Introduction

Unusually short body stature, known as dwarfism, is a condition of growth deficiency and reduced body weight caused by a variety of hereditary and hormonal disruptions. In humans, different dwarf syndromes have been characterized by genetic mutations affecting a wide range of genes (Godowski et al., 1989; Mortier et al., 2000; Richette et al., 2008). Various short body statures have also been investigated in animal species including dogs, cattle, pigs, and chickens (Boegheim et al., 2017). Dwarf animals in native or commercial breeds, have held the interest of humans over the past century, and have been bred for either ornamental or economic reasons. For example, plenty of distinct dwarf phenotypes have been described and studied in the chicken literature (Hutt, 1949). One of the best-studied hereditary variations in growth deficiency is sex-linked dwarfism, which is a proportional dwarfism, caused by the mutation in the *GHR* (Burnside et al., 1991; Agarwal et al., 1994).

Autosomal dwarfism (adw) in chicken is a recessive trait resulting in reduced body weight with short stature, despite the normal hormonal concentration of GH and insulin-like growth factor 1 (IGF1). The locus responsible for chicken *adw* was first, and uniquely, found in a Cornell K-strain of White Leghorns, and shown to be caused by a recessive single locus (Cole and Hutt, 1973). The overall appearance of autosomal dwarf chicken was recognized as a small body stature by short shank length, with 30–40% reduced adult body weight (Cole and Hutt, 1973; Cole, 2000). Investigations into known hormonal factors related to body growth showed no significant decrease in the plasma concentrations of GH and IGF1 compared with normal animals, which is distinct from other types of dwarfisms (Scanes et al., 1983; Huybrechts et al., 1985). Only a slight decrease in plasma thyroid hormones T3 and T4 was observed (Lam et al., 1989). Therefore, adw is not considered to be determined by a direct genetic aberration in the GH-IGF1 molecular pathway, which is known to determine body size in other vertebrates (Yakar et al., 2002). While the metabolic and genetic factors underlying adw have been studied (Scanes et al., 1983; Huybrechts et al., 1985; Ruyter-Spira et al., 1998a), the causative variation determining the autosomal dwarf locus have remained elusive. The *adw* locus was mapped to chromosome 1, by using bulked segregant analysis with microsatellite markers (Ruyter-Spira et al., 1998a). In that study, genotype frequencies of markers were evaluated respectively from affected and unaffected individuals in the autosomal dwarf segregating population. The *adw* locus is closely linked to microsatellite marker LEI0146 on chromosome 1, with a recombination fraction of 0.03 and LOD score of 31.98 (Ruyter-Spira et al., 1998a). Two potential candidate genes were harbored in the region associated; the gene coding for the high-mobility group protein I-C (*HMGI-C*), an alias for *HMGA2*, and *IGF1* (Ruyter-Spira et al., 1998a). A continuation of this research showed no mutations in the coding sequence of these two genes, and no difference in expression of *HMGA2* (Ruyter-Spira et al., 1998b). With the whole-genome sequence techniques, we are now able to explore the *adw* genome. In the present study, we aimed to identify the *adw* associated region and the potential causal variant underlying the autosomal dwarf phenotype.

## Materials and Methods

### Animals Used for Sequencing and Genotyping

To identify the variant underlying adw, we performed the whole-genome sequencing on a 6-day homozygous embryo (*adw/adw*), collected from the mating dwarf parents. For candidate validation; we genotyped two 6-day and one 8-day old dwarf embryos (*adw/adw*), as well as two pooled sperm samples from 20 Cornish and 20 White Leghorn adw cocks respectively. In addition, two normal-sized embryos; 6 and 8 days of age respectively, were used as controls for genotype and expression analyses. The dwarf samples we used for this study have no genetic relationship.

### Whole-Genome Sequencing and Variant Calling

Whole-genome sequence data were generated for the embryos of autosomal dwarf and normal-sized chickens. We sequenced the genomic DNA by Illumina HiSeq 2000 paired-end sequencing (2 × 100 bp read length). The sequence reads were then trimmed for quality using Sickle (Joshi and Fass, 2011). The software BWA-MEM (version 0.7.15) (Li and Durbin, 2009) was used to map the clean reads to the Red Jungle fowl genome assembly, with the build Gallus_gallus-5.0 (International Chicken Genome Consortium, 2015). Duplicate reads were removed by using Samtools dedup function (Li et al., 2009). Local realignment of the reads around small indel was done using GATK IndelRealigner (McKenna et al., 2010). We performed variant calling by using Freebayes with sound settings (-min-base-quality 10, -min-alternate-fraction 0.2, -haplotype-length 0, -pooled-continuous, -min-alternate-count 2) (Garrison and Marth, 2012). Variant filtering was conducted toward the SNPs and indels using VCFtools (Danecek et al., 2011), with a mean depth between 3 and 25, and genotype call rate > 0.7. In addition, we used LUMPY to identify the structural variants, with the default settings for split-read detection (Layer et al., 2014). Functional annotation for all types of detected variants was generated by Variant Effect Predictor (VEP) (McLaren et al., 2016).

### Mapping of adw Candidate Variant by Sequence Comparison

We used the previous linkage data to construct the candidate region. In the adw linkage study published by Ruyter-Spira et al. (1998a), the *adw* locus was mapped 3 cM downstream of marker LEI0146 (NC_006088.4:g.53,274,224_53,274,474). The average linkage resolution on chromosome 1 is around 0.3–0.4 Mb/cM (Groenen et al., 2009), which suggests the most likely location of *adw* is at around 54.1–54.4 Mb. Because of the mapping uncertainty, we used a margin of 2 Mb around this location and therefore extended the candidate region from 52 to 56 Mb, which harbors *IGF1* for subsequent investigation. We used sequence data within the candidate interval from 261 White Leghorns as controls. We compared the genetic variants, including SNPs and indels, of the *adw/adw* chicken against the normal-sized controls by using VCFtools. We compared the allele at each position, and focused on the unique allele carried by adw for subsequent fine mapping. Schematic representations of the candidate region were visualized using Gviz (v1.22.2) (Hahne and Ivanek, 2016) with ideogram track and the gene of interest. For homozygosity analysis, we calculated the runs of homozygosity (ROH) to estimate autozygosity for the sequenced adw individual. ROHs for an individual were calculated based on the following criteria specified in Bosse et al. (2012). This included the number of SNPs, in a window size of 10 Kb, counted below 0.25 times the average whole-genome SNP count; and the homozygous stretches contained at least 10 consecutive windows which showed a total SNP average lower than the genomic average. Sufficiently covered windows with 0.5–2 times the average depths were taken into account. The relaxed threshold for individual windows were used within a homozygous stretch to avoid local assembly or alignment errors, which was done by allowing for maximum twice the genomic average SNP count, and the average SNP count within the candidate ROH to not exceed 1/4 the genomic average.

Fine mapping inside the candidate region was conducted by a four-step filtering procedure. In step 1, at each position, we compared unique variants that are only present in the adw dataset, and absent in the normal-sized White Leghorns. Autosomal dwarfism, inherited as a recessive trait, is expected to be homozygous for the *adw* locus. Therefore, in step 2, heterozygous sites were filtered out, and only the *adw* homozygous sites were kept for further analyses. In step 3, we filtered variants based on the consequence annotation. We predicted the variant consequence with the uniform terms defined by Sequence Ontology (Eilbeck and Lewis, 2004), using VEP (McLaren et al., 2016). The consequence terms evaluate the effect that each substitution may have, based on their properties on different transcripts, with more than one variant consequence. To help categorize the impact of the variants, we assessed the consequence terms with four impact categories; high, moderate, low, and modifier, by using SnpEff (Cingolani et al., 2012). We split the variant list into two groups: one containing variants with high and moderate impact, which likely alter protein structure and function; another list with variants of low to modifier impact, which are mostly non-coding variants. We investigated the expression among tissues of long intergenic non-coding RNAs (lincRNAs) in the list, the quantification of each lincRNAs was calculated and retrieved from two baseline transcriptome studies on Expression Atlas (Barbosa-Morais et al., 2012; Merkin et al., 2012; Petryszak et al., 2016, 2017). The missense variants were predicted with SIFT score (Sim et al., 2012), to estimate the alteration of the amino acid and the degree of conservatism among species. The SIFT score defines a missense variant as “deleterious” (0–0.05) or “tolerated” (0.05–1). In step 4, we screened the public sequence and SNP repositories for novel variants. The *adw* mutation is not segregating within other population, hence variants identified in these other populations were excluded to ensure that potential causative mutations are not harbored by normal-sized population.

### Validation of the adw Mutation

Based on the fine mapping steps outlined above, three adw chicken embryos and two mixed sperms were genotyped to validate the candidate mutation. We performed genotyping for the mutation NM_001006244.1:c.433G > A on chromosome 1 in transmembrane protein 263 (*TMEM263*) by polymerase chain reaction-restriction fragment length polymorphism (PCR-RFLP) analysis. We genotyped both dwarf (*adw/adw*) and normal-sized (*ADW/ADW*) chickens. The mutation in *TMEM263* was amplified with the following primers; TMEM263_1F: 5′-GTTCAATCAAAGACCACCCG-3′; TMEM 263_1R: 5′-TTGGCTTTAGTCAGACTTGTCCT-3′. The PCR products were then digested with the enzyme *Ddel* (New England Biolabs, United Kingdom) and analyzed by 3% agarose gel electrophoresis, the adw samples are expected to include fragments of 109 and 184 bp, while the normal-sized ones are expected with fragments of 294 bp.

### Candidate Gene Expression Analyses

We estimated the gene expression difference between the dwarf and normal-sized chicken for *TMEM263* and *IGF1*, using Reverse Transcription quantitative PCR (RT-qPCR). RNA was extracted from the same two 6-day and one 8-day old embryos used in genotyping, from which cDNA was synthesized. Three normal-sized embryos (*ADW/ADW*) of 6 and 8 days old respectively, were used as controls. We conducted three technical replicates for each sample. The expression differences were determined by the ΔΔCt method using 28S rRNA as a housekeeping gene for normalization. Target sequences were amplified from cDNA with the following primers, TMEM263_2F: 5′-GCCACCAGAAGGTTCAATCAAAG-3′; TMEM263_2R: 5′-CTGAAGATGCCACCAGTCACA-3′; IGF1_F: 5′-CTTGAAGGTGAAGATGCACACTG-3′; IGF1_R:5′-GGCAGCAGCAGAACTGGTTA-3′. The RT-qPCR was performed on an Applied Biosystems^®^ 7500 Real-Time PCR Systems, using MESA Blue qPCR Mix Plus for SYBR Assay (Eurogentec, Belgium).

### Functional Comparative Analyses of Orthologous Genes

As the function of TMEM263 protein in chicken is poorly characterized, we used the orthologous gene to investigate the potential function. A multi-species alignment of TMEM263 was generated by Clustal Omega (v1.2) (Sievers et al., 2014). The orthologous proteins of TMEM263 aligned were obtained from 10 representative vertebrates (*D. rerio, H. sapiens, P. troglodytes, M. mulatta, B. taurus, C. lupus, M. musculus, R. norvegicus, X. tropicalis*, and *G. gallus*) at NCBI HomoloGene. We reconstructed a Neighbor-Joining Tree of the TMEM263 proteins with zebra fish as the outgroup, and visualized the result using Jalview (v2.10.3) (Waterhouse et al., 2009). The secondary topology of the transmembrane domain of TMEM263 was predicted by TMHMM Server (v. 2.0) based on a hidden Markov model (Krogh et al., 2001). The predicted protein feature was displayed using Protter 1.0 (Omasits et al., 2014). We could not predict the three-dimensional structure in the study, due to low coverage (<50%) and low confidence (<20%) of the prediction. With the human orthologous gene, a gene network analysis was generated by using the association network predicting server GeneMANIA (Zuberi et al., 2013). Based on the function and properties of human *TMEME263*, the program explored the functional associated gene networks in genomic and proteomic data sources. Each network is weighted by the corresponding data source based on Gene Ontology biological process co-annotation, and with label propagation algorithm, which represents how well the genes are connected and interacted with each other.

## Results

### Identification of the Candidate Causal Mutation for Autosomal Dwarfism

**FIGURE 1:**
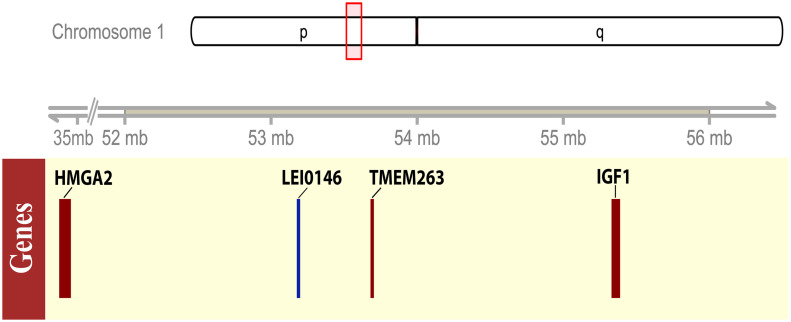
Schematic representation of the candidate region for *adw* locus on chromosome 1. The upper panel showed the ideogram of the chicken chromosome 1, the candidate region, 52–56 Mb, was shown by a red box on the ideogram tracks. The lower panel showed the gene model by the solid box, indicating the size of the coding region of the representative candidate genes or the microsatellite marker. The associated microsatellite marker LEI0146 was plotted in blue, while the genes with interest, *HMGA2, TMEM263, IGF1*, presented in red boxes.

To study the causal variants that underlie adw, we investigate the whole-genome sequence data of an adw chicken. The adw sequence data was aligned to the reference genome (Gallus_gallus 5.0), which resulted in an average coverage of 9.1x. Linkage analyses previously located the *adw* locus downstream microsatellite marker LEI0146 at around 54 Mb. Therefore, the gene *HMGA2* studied by Ruyter-Spira et al. (1998a,b) was mapped on the current reference genome at position 34 Mb, and was not considered as a candidate in the present study. ROH analysis for chromosome 1 showed an ROH of 14.8 Mb, spanning the region from 51.2 to 65.9 Mb, which is expected to be identical by descent. Although larger, the ROH overlaps with the location of the candidate region (Supplementary Figure S1) most likely as a consequence of the strong selection for the *adw* haplotype in the chicken lines used. The candidate region we defined, spanned position 52–56 Mb on chromosome 1 (**Figure 1**). Within the candidate region, we found 146,070 variants, including genic variants in 72 genes and intergenic variants; we did not observe any specific structural variants that could contribute to growth reduction, as they were not located in the coding region. Supplementary Table S1 showed the structural variation identified in the candidate region.

**FIGURE 2:**
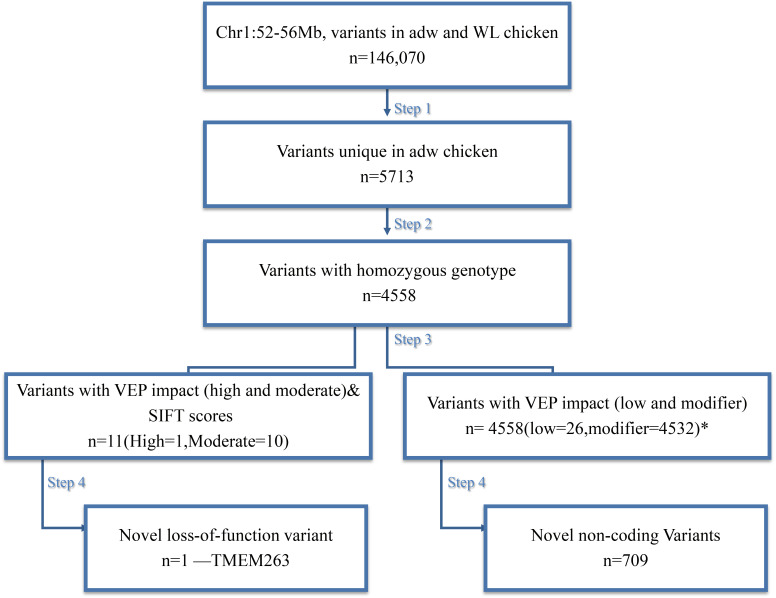
Schematic representation of the four steps filtering to identify the candidate variant for the chicken adw locus. n = number of variants after each filtering step. Autosomal dwarfism is represented by adw, while WL is the White Leghorn Chickens. After the filtering, there is one candidate SNP exclusively found in adw and with a high impact on the consequence, shown at the left path. While there is a list of variants also uniquely fond in the adw chicken, they are mainly located on the non-coding region, shown at the right path. During variant impact prediction, each variant could have more than one impact assigned, as the impact estimation was based on different transcript. ^∗^Note that among these variants, 11 of them contain more than one impact. The sum of variant numbers therefore is not consistent with the result from the last step.

We performed a four-step filtering procedure for all the variants identified by variant calling (**Figure 2**). After comparing the variants in the dwarf and normal-sized chickens in step 1, we generated a set of 5,713 variants in the candidate region that are unique for adw. Because adw is a recessive trait, we removed all heterozygous sites seen in the dwarf chicken; after step 2, 4,558 homozygous variants remained. In step 3, homozygous variants were predicted for substitution impact by VEP and SIFT, resulting in a list of 11 variants with moderate to high impact (**Table 1**), and a list of 4,558 variants within non-coding regions, all with low and modifier impact. At step 4, of the variants list with moderate to high impact, we further excluded 10 SNPs by screening the NCBI database, as they were not unique or exclusive for the adw chicken. The final candidate causal mutation is a nonsense variant in *TMEM263* gene (NM_001006244.1:c.433G > A). It is the only mutation with a high consequence impact that results in a loss-of-function alteration in the transmembrane protein 263. Among the non-coding variants, 709 variants in the dataset were shown to be absent from the dbSNP database (Supplementary Table S2). Of these variants, 38 SNPs are located in six known lincRNAs. Two baseline transcriptome studies show the expression of the six lincRNAs in chicken tissues, which are ubiquitously expressed (Supplementary Figure S2).

**Table 1:** Variants uniquely found in autosomal dwarf chicken and completely associated with dwarfism.

Position	Reference dbSNP	Ref/Alt	Gene	Variants	Impact	Database
52195787	rs736218372	TC/CT	HMGXB4	Missense variant	Moderate	NCBI
53369104	rs14825651 and rs14825651	AA/TT	ASCL4	Missense variant and splice region variant	Moderate	NCBI
53369406	rs741302250	G/A	ASCL4	Missense variant (deleterious)	Moderate	NCBI
53376828	rs733697531	C/T	PRDM4	Intron variant	Moderate	NCBI
53380585	rs313232660	G/A	PRDM4	Missense variant	Moderate	NCBI
53683208	rs15270486	A/G	MTERF2	Missense variant	Moderate	NCBI
53688583	N.A.	C/T	TMEM263	Stop gained	High	N.A.
54232753	rs732172030	G/A	C12orf75	Missense variant	Moderate	NCBI
54834890	rs733216275	AT/GA	STAB2	Downstream gene variant and missense variant (deleterious)	Moderate	NCBI
54838941	rs14827457 and rs14827458	TG/CC	STAB2	Missense variant and upstream gene variant	Moderate	NCBI
55461367	rs13869828	A/G	PARPBP	Missense variant	Moderate	NCBI


**FIGURE 3:**
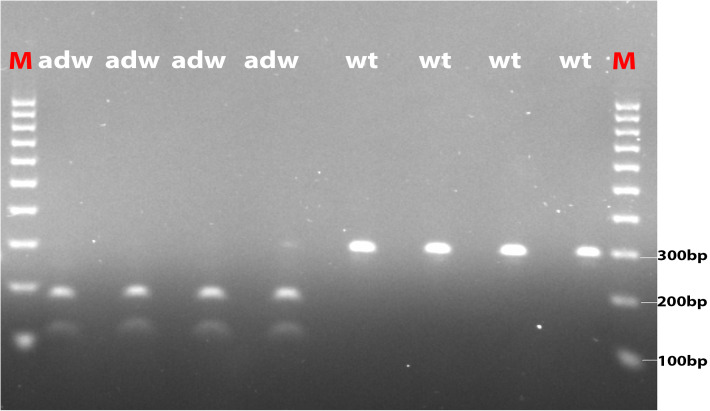
The photo of agarose gel of amplified fragments for genotyping candidate mutation in *TMEM263*. Autosomal dwarf (adw) and normal-sized (wt) samples were analyzed by PCR-RFLP method. Amplified DNA was digested with *Ddel* and separated by electrophoresis. Markers were used in both end lanes (M). The autosomal dwarf samples (adw/adw) were shown with two sequence fragments, around 109 and 184 bp, respectively, while the wild-type samples shown with one band on the right four lanes, the size is around 294 bp.

The nonsense mutation in *TMEM263* causes premature termination of the protein, leading to a truncation of the transmembrane protein. The mutation alters the 59^th^ amino acid residue from tryptophan (Trp) to a stop codon (^∗^) at the first membrane-spanning helix. The alteration truncates the protein to 50% and was expected to essentially affect the protein function. We validated the loss-of-function mutation in *TMEM263* using PCR-RFLP in all autosomal dwarf samples. The five autosomal dwarf samples indeed showed homozygous for the variant allele, whereas all normal-sized controls do not carry the mutation (**Figure 3**). The NP_001006244.1:p.(Trp59^∗^) variant in *TMEM263* was identified to be the most interesting candidate causal mutation. The RT-qPCR revealed that the expression level of *TMEM263* and *IGF1* mRNA showed no significant difference between normal and dwarf individuals (Supplementary Figure S3).

### Functional and Comparative Analyses of the Candidate Gene

**FIGURE 4:**
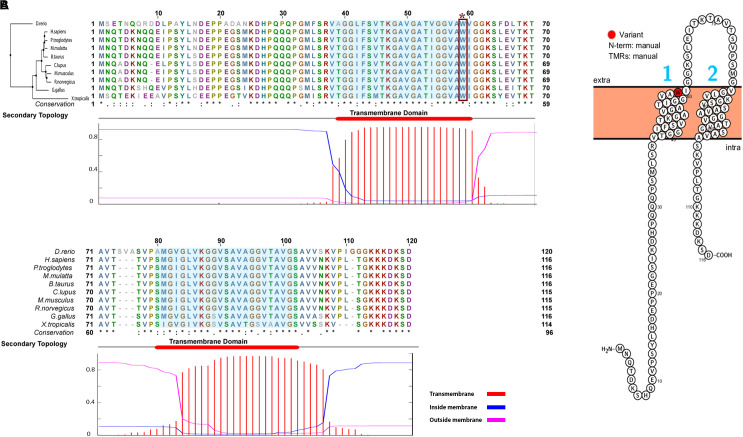
Comparative analyses and the predicted topology of TMEM263. **(A)** Multi-species alignment of orthologous TMEM263 protein sequence. The Neighbor-Joining tree of TMEM263 is at the top left side of the alignment, sequence of zebra fish (*D. rerio*) was used as outgroup. The upper panel shows the alignment of TMEM263 protein, the bottom sequence represents the conservation of the each amino acid. The asterisk (^∗^) indicates position which has a single, fully conserved residue. The period (.) indicates conservation between groups of weakly similar properties, and the colon (:) indicates strongly similar conservative properties. The lower panel is the prediction of the transmembrane domains, shows the location and the orientation of transmembrane domains in the TMEM263 sequence. The x-axis refers to the location of amino acid residue sites; y-axis represents the probability of topology for each region. The predicted transmembrane region (TMR) is shown in red (amino acid residue sites: 38–60; 80–102), blue lines stand for intracellular region, and the magenta line is the amino acid predicted outside the membrane. The consensus line is placed at the top of the plot. The transmembrane domains are highlighted in sequence alignment with light blue background. The nonsense mutation (Trp59^∗^) is demonstrated by dark red rectangle. **(B)** The predicted topology of TMEM263 protein. The topology was illustrated with the position of the variation shown in red. The N-terminal location and the TMRs were generated based on the topology prediction, and input manually. The numbers 1 and 2 in blue refer to the two TMRs, the cell membrane is in light orange, and N-term is predicted on the cytoplasmic side of the membrane.

**FIGURE 5:**
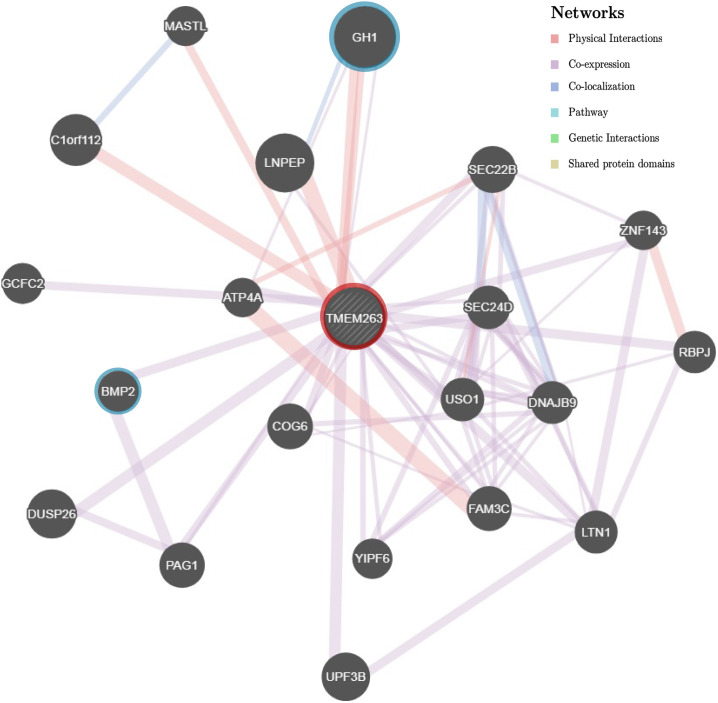
Gene association network of human *TMEM263* gene. The gray dashed circle in the center with red ring represents TMEM263. The size of the solid circles representing other genes is proportional to the number of interactions they have. Gene *GH1* and *BMP2* are highlighted with blue rings to emphasize the two network branches. Red branch stands for physical interaction, purple branch displays co-expression, and blue branch means co-localization.

The *TMEM263* gene is highly conserved across various species, from primates to craniata (**Figure 4A**). The protein sequence identity of orthologous *TMEM263* genes between chicken and other vertebrates ranges around 73–89%. The comparison with human homologous protein presented the conservation around 89%. The tryptophan amino acid site at position 59, which harbors the nonsense mutation, is present fully conserved across all species analyzed. The predicted topology of the vertebrate TMEM263 protein is a multi-pass membrane structure, which consists of two transmembrane domains (from amino-acid positions 38 to 60, and from 80 to 102), with the nonsense mutation located at the first transmembrane region (TMR) (**Figure 4B**). Based on human orthologous genes, we generated a gene network analysis to investigate the potential function of *TMEM263*. Interestingly, TMEM263 was shown to have physical interactions with growth hormone (*GH1*), and to be co-expressed with bone morphogenetic protein (*BMP2*) in human cells (**Figure 5**).

## Discussion

In this study, we fine-map and report the likely causal variant of the chicken adw locus, located on chromosome 1, by comparing whole-genome sequence data of an autosomal dwarf with normal-sized chickens. Given the origin of adw in chickens, emerging from the Cornell White Leghorn strain, we used normal-sized White Leghorn chickens as the control in this study. Moreover, the causal variant for adw is unlikely to be segregating in other non-dwarf populations. The linkage results show the strong association between *adw* locus and microsatellite marker LEI0146, which defined a 4 Mb surrounding interval on chromosome 1 as the candidate region. The ROHs analyses show low SNP abundance in the candidate region, and overlaps with the location of the candidate region. The candidate region was chosen to include *IGF1*, known to be functionally associated with growth traits in humans (Yakar et al., 2002). As endocrinological study revealed unaffected IGF1 concentration in adw chickens (Ruyter-Spira et al., 1998b), as well as the unaffected differential expression we identified, the IGF1 was not considered the best candidate of the study. After screening the variants, only one novel nonsense mutation in *TMEM263* remained as a strong candidate and proven associated with the autosomal dwarf phenotype.

In an autosomal dwarf dataset, we found, in total, 710 variants in the candidate region are associated with the adw phenotype, including a nonsense mutation in gene *TMEM263* likely underlying the adw phenotype. The gene *TMEM263* encodes for a transmembrane protein, but its function in chicken is not well-known. Meanwhile, the 709 variants of lower impact fall within 58 genes and six known lincRNAs. The genic variants with lower impact in the list are mostly located within the non-protein coding region, containing intron and UTR, and are assumed not to disrupt the function of these genes. The six lincRNAs in the candidate region are barely studied, their ubiquitous expression in chicken tissues makes it hard to predict the contribution of these lincRNAs (Barbosa-Morais et al., 2012; Merkin et al., 2012; Petryszak et al., 2017). Due to the limited annotation for non-coding variants in the reference chicken genome, detailed studies on those variants as causal mutation is less favorable in this study. In summary, it is unlikely, although still possible, that the 709 variants harbor the causal mutation; therefore we consider these variants as less potential candidates for *adw* mutation.

The *TMEM263* protein is conserved across species, and the transmembrane protein sequence belongs to a conserved protein domain (model UPF0444, pfam15475). This domain is a conserved unit from a superfamily of proteins representing multi-pass membrane structure, including two helical membrane-spanning domains. The membrane-spanning domains are located between amino acid positions 38–60 and 80–102. The protein consists of an intracellular N- and C-terminus, along with an extracellular loop. The nonsense mutation is uniquely found in adw chicken in the first transmembrane-spanning domain, and is completely associated with the autosomal dwarf phenotype. The expression level of *TMEM263* in 6–8 day embryos does not differ between dwarf and non-dwarf chickens. In two baseline transcriptome experiments, *TMEM263* expressed ubiquitously in chicken tissue, including skeletal muscle tissue (Barbosa-Morais et al., 2012; Merkin et al., 2012). As a consequence of the nonsense mutation, the transmembrane protein is truncated and only contains the N-terminal amino acids 1–58. Accordingly, the altered transmembrane protein loses part of the conserved domain UPF0444, as well as one of the membrane-spanning domains, almost certainly resulting in protein function loss.

Although little is known about the role of the *TMEM263*, evidence from studies in humans implied that *TMEM263* (former alias: *C12orf23*) is associated with growth and bone development (Ewing et al., 2007; Estrada et al., 2012; Calabrese et al., 2017). In a human Genome-Wide Association Study, *TMEM263* was shown to be significantly associated with femoral neck bone mineral density (FN-BMD) (Estrada et al., 2012). The expression level of *TMEM263* was also correlated with BMD and osteoporotic fracture risk. A cortical bone co-expression network study showed the expression of *TMEM263* to be significantly correlated with the osteoblast functional modules (OFMs), which impacts bone mineral density by altering the activity of bone-forming osteoblasts (Calabrese et al., 2017). Considering the strong positive correlation between bone mass and bone size, the co-expression between *TMEM263* and OFM indicates that *TMEM263* is likely functionally involved in cartilage and bone formation. In addition, a large-scale human disease study reported that *TMEM263* physically interacts with growth hormone 1 (Ewing et al., 2007), an important regulation somatotropin in growth development. In the protein-protein interaction study, TMEM263 was identified by co-immunoprecipitation with GH1 followed by mass spectrometry. Therefore, TMEM263 may be involved in the growth pathway by potentially acting as a regulator in transport or signal transduction. *TMEM263* was also identified as an interaction partner of potassium channel genes *Slick* and *Slack*, which is a sodium-activated channel widely expressed in the central nervous system (Rizzi et al., 2015). Taken together, these studies in humans suggest a potential role of *TMEM263* in skeletal development, like cartilage and bone formation. This supports our hypothesis that the loss-of-function mutations in gene *TMEM263* will likely influence the deposition of bone mineral, thereby affecting skeletal development and body growth, which were observed in autosomal dwarf chickens.

## Conclusion

We describe a novel nonsense mutation on chromosome 1, located in the highly conserved gene *TMEM263*, which is associated with chicken *adw*. The adw associated NP_001006244.1:p.(Trp59^∗^) mutation in the TMEM263 protein was expected to functionally affect the protein and exclusively found in adw chicken. The novel premature termination is the most likely variant that underlying the chicken adw. Functional information in humans supports its potential role in bone development and growth. Our results suggest a potentially vital role of *TMEM263* in growth reduction and provide the basis for future systematically verifying the function of the transmembrane protein 263.

## Data Accessibility

The sequence data of autosomal dwarf chicken was deposited in ENA, under the primary Accession No. PRJEB25937, sample ERS2374285. The sequence data within the 4 Mb candidate region used in the study is available upon the request.

## Author Contributions

MG and RC conceived and designed the study. ZW analyzed the data and wrote the manuscript. MD helped with data analysis. BD performed the experiments. MD, H-JM, RC, and MG provided valuable suggestion and comments to improve the manuscript. All authors have read and approved the final manuscript.

## Disclaimer

No ethics statements were required during samples recording according to the Dutch ethical policy on animal protection and welfare(Gezondheids- en welzijnswet voor dieren).

## Conflict of Interest Statement

The authors declare that the research was conducted in the absence of any commercial or financial relationships that could be construed as a potential conflict of interest.
