# Corrigendum: A novel loss-of-function variant in transmembrane protein 263 (TMEM263) of autosomal dwarfism in chicken

**DOI:** 10.3389/fgene.2023.1349789

**Published:** 2024-01-08

**Authors:** Zhou Wu, Martijn F. L. Derks, Bert Dibbits, Hendrik-Jan Megens, Martien A. M. Groenen, Richard P. M. A. Crooijmans

**Affiliations:** Animal Breeding and Genomics, Wageningen University & Research, Wageningen, Netherlands

**Keywords:** autosomal dwarfism, body size, recessive trait, chicken, loss-of-function mutation

In the published article, there was an error. We discovered an error in the description of the primers used in the study.

A correction has been made to **Materials and Methods**, Validation of the adw Mutation, Paragraph 1. This sentence previously stated:

“The mutation in *TMEM263* was amplified with the following primers; TMEM263_1F: 5′-AGG​TTC​AAT​CAA​AGA​CCA​CCC​G-3′; TMEM263_1R: 5′-CCC​GTT​AAA​GGC​ACT​TTG​CT-3′.”

The corrected sentence appears below:

“The mutation in *TMEM263* was amplified with the following primers; TMEM263_1F: 5′-GTT​CAA​TCA​AAG​ACC​ACC​CG-3′; TMEM263_1R: 5′-TTG​GCT​TTA​GTC​AGA​CTT​GTC​CT-3′.”

In the published article, there was an error. We discovered there is a printing mistake in the **Introduction** regarding the description of sex-linked dwarfism.

A correction has been made to **Introduction**, Paragraph 1. This sentence previously stated:

“One of the best-studied hereditary variations in growth deficiency is sex-linked dwarfism, which is a disproportional dwarfism, caused by the mutation in the GHR (Burnside et al., 1991; Agarwal et al., 1994).”

The corrected sentence appears below:

“One of the best-studied hereditary variations in growth deficiency is sex-linked dwarfism, which is a proportional dwarfism, caused by the mutation in the GHR (Burnside et al., 1991; Agarwal et al., 1994).”

The authors apologize for these errors and state that these do not change the scientific conclusions of the article in any way. The original article has been updated.

