# Supplementary figures and images for: A Novel Loss-of-Function Variant in Transmembrane Protein 263 (TMEM263) of Autosomal Dwarfism in Chicken
Source: Front Genet. 2018 Jun 5;9:193. doi: 10.3389/fgene.2018.00193 (PMC6001002; doi:10.3389/fgene.2018.00193)

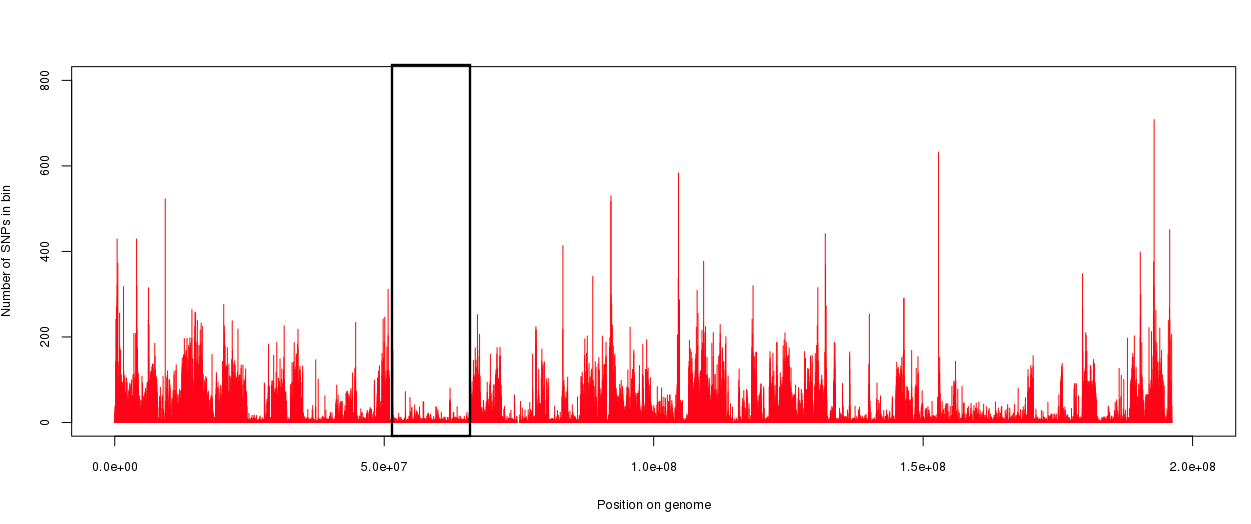

Supplement: Supplementary file 1 [file Image_1.PNG]

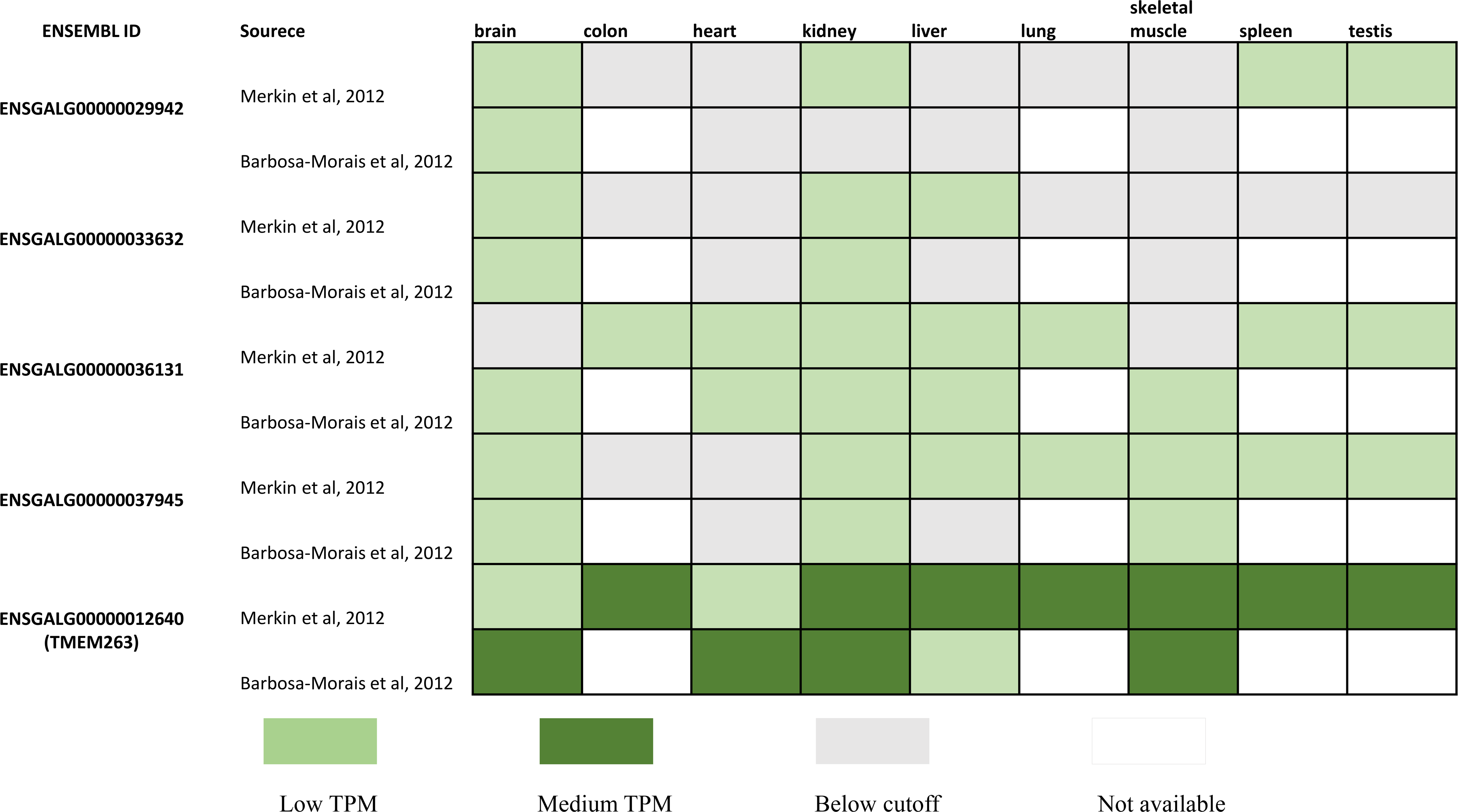

Supplement: Supplementary file 2 [file Image_2.TIF]

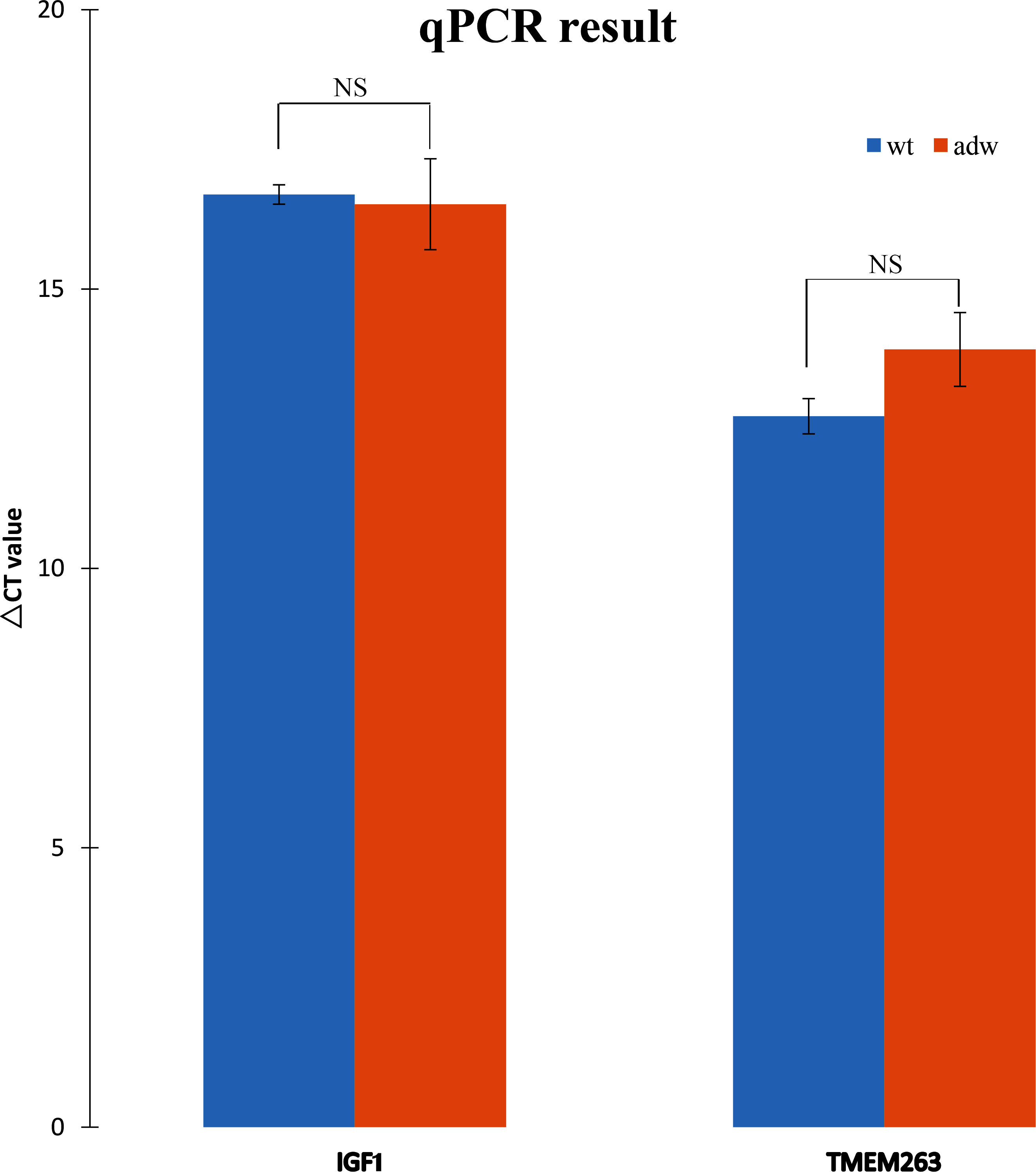

Supplement: Supplementary file 3 [file Image_3.TIF]
