# Supplementary material for: A Novel Loss-of-Function Variant in Transmembrane Protein 263 (TMEM263) of Autosomal Dwarfism in Chicken
Source: Front Genet. 2018 Jun 5;9:193. doi: 10.3389/fgene.2018.00193 (PMC6001002; doi:10.3389/fgene.2018.00193)
Supplement: Supplementary file 6 [file Data_Sheet_1.pdf]

Supplementary Figure Legends

Figure S1. ROHs analyses and the distribution of nucleotide diversity over chromosome The x-axis displays the physical position on the chromosome and the y-axis shows the corrected number of SNPs that was called in bins of 10 kb. The black rectangle illustrates the homozygous stretch, located around 51.0 Mb to 65.9 Mb, supporting to the candidate region of autosomal dwarfism.

Figure S2. Baseline Expression of the lincRNA and TMEM263 gene. Of the variants of low to modifier impact, there are 6 lincRNAs have been annotated, among them 4 have the public transcriptome data from two studies, in which expression was tested in untreated conditions. The Transcripts Per kilobase Million (TPM) of several tissues were shown in green color. Tissues have TPM value between 0.5 to10 are recognized as low TPM, and TPM from 11 to 1000 represents medium expression level, gray and white boxes show the tissues without a valid expression data.

Figure S3. Expression of IGF1 and TMEM263 among normal-sized and autosomal dwarf chickens. Figure shows the qPCR result of gene TMEM263 and IGF1. There is no significant difference in the △CT value between normal-sized (blue) and autosomal dwarf (red) chickens.

Supplementary Table Legends

Table S1. Structrual Variations detected in the autosomal dwarf chicken.

Table S2. Uniqe variants of low to moderate impact in autosomal dwarf chicken.
